# COGNITIVE DISCREPANCY ON SOCIAL LIFE AND LIFE SATISFACTION AMONG OLD ADULTS WITH VISUAL DISABILITIES

**DOI:** 10.1093/geroni/igae098.2682

**Published:** 2024-12-31

**Authors:** Ya-Han Chang, Silvia Sörensen

**Affiliations:** University of Rochester, Rochester, New York, United States; University of Rochester, Rochester, New York, United States

## Abstract

**Background and objectives:**

This study addresses the quality of life faced by individuals with vision impairment. Life satisfaction in the context of disability is co-determined by social life, loneliness feelings, future planning, and financial status. However, the role of cognitive discrepancy and social cognition congruence in shaping life satisfaction is rarely studied. This study uncovers the role, by investigating various factors, including social relationships, loneliness, and discussions of future care plans.

**Research Design and Method:**

Baseline data from older adults with age-related macular degeneration (N=167, Mean age = 79.66), who were participants in a depression prevention trial, were analyzed to determine the relationship between future care planning to life satisfaction. Cognitive discrepancy, and moderation of this relationship by variable (1) loneliness and (2) quality of social relationship tested.

**Results:**

The significant relationship between discussions about future care plans and older adults’ life satisfaction (p <.001) was moderated by the interaction of the quality of social relationships and loneliness as either cognitive discrepancy or congruence.

**Discussion and Implication:**

The pivotal role of social cognitive congruence about loneliness and social relationships emerges as a critical factor in life satisfaction among old adults. We found the influence of cognitive discrepancy and care plan discussion on life satisfaction. Discussing plans with family members for elders with high cognition discrepancies is critically affecting their life satisfaction.

